# An ECG Signal Classification Method Based on Dilated Causal Convolution

**DOI:** 10.1155/2021/6627939

**Published:** 2021-02-02

**Authors:** Hao Ma, Chao Chen, Qing Zhu, Haitao Yuan, Liming Chen, Minglei Shu

**Affiliations:** ^1^Shandong Artificial Intelligence Institute, Qilu University of Technology (Shandong Academy of Sciences), Jinan 250353, China; ^2^Qilu Hospital of Shandong University, Jinan 250012, China; ^3^Department of Cardiology, Shandong Provincial Hospital Affiliated to Shandong First Medical University, Jinan, Shandong 250021, China

## Abstract

The incidence of cardiovascular disease is increasing year by year and is showing a younger trend. At the same time, existing medical resources are tight. The automatic detection of ECG signals becomes increasingly necessary. This paper proposes an automatic classification of ECG signals based on a dilated causal convolutional neural network. To solve the problem that the recurrent neural network framework network cannot be accelerated by hardware equipment, the dilated causal convolutional neural network is adopted. Given the features of the same input and output time steps of the recurrent neural network and the nondisclosure of future information, the network is constructed with fully convolutional networks and causal convolution. To reduce the network depth and prevent gradient explosion or gradient disappearance, the dilated factor is introduced into the model, and the residual blocks are introduced into the model according to the shortcut connection idea. The effectiveness of the algorithm is verified in the MIT-BIH Atrial Fibrillation Database (MIT-BIH AFDB). In the experiment of the MIT-BIH AFDB database, the classification accuracy rate is 98.65%.

## 1. Introduction

According to the “China Cardiovascular Disease Report 2018” [1], the prevalence of atrial fibrillation (AF) in China is on the rise, and the mortality rate has long been higher than that of tumors and other diseases. Since most cardiovascular diseases are not isolated diseases and there are no significant clinical features in the early stage, a large number of cardiovascular disease patients have related complications during the initial diagnosis, greatly threatening their health. Besides, the actual prevalence of cardiovascular disease may be much higher than the estimated level; therefore, the timely and accurate detection of cardiovascular disease is of great significance.

The electrocardiogram (ECG) examination has become one of the four major routine examination items in modern medicine. ECG is the safest and most effective method for diagnosing cardiovascular diseases. The rapid development of electronic information technology has made ECG measurement more convenient and faster, which provides a lot of data for ECG automatic classification.

The theory of deep learning was proposed in the 1940s, but due to limited computing power, its development was particularly slow. After the 21st century, with the rapid development of computer technology and parallel accelerated computing technology, deep learning has been supported by hardware. In 2012, Hinton's research team participated in the ImageNet image recognition competition, and the AlexNet [2] built by convolutional neural networks(CNNs) won the championship, which attracted the attention of academia and industry to the field of deep learning. Rajpurkar et al. [3] constructed a 34-layer CNNs and verified the effectiveness of the network in a self-built database and compared it with the conclusions given by medical experts. The final F1 score of the neural network is 77.6%, which is higher than the 71.9% of medical experts. In the MIT-BIH Atrial Fibrillation Database (MIT-BIH AFDB) [4]. He et al. [5] used continuous wavelet transform (CWT) to convert the ECG signals into a spectrogram and then used CNNs to automatically extract features and classify them. The final classification accuracy was 99.23%. Wang et al. [6] used the wavelet packet transform and random process theory to extract features and used artificial neural networks (ANNs) for classification. The classification accuracy rate was 98.8%. Lake et al. [7] used the coefficient of sample entropy (COSEn) to classify atrial fibrillation signals. Asgari et al. [8] use wavelet transform to process the signals and use a support vector machine (SVM) to detect the occurrence of atrial fibrillation. Zhou et al. [9] used a new recursive algorithm to classify atrial fibrillation signals. Acharya et al. [10] use 11-layer CNNs to detect the occurrence of atrial fibrillation. Andersen et al. [11] combine CNNs with RNNs and use RR intervals to strengthen the network classification capabilities. Dang et al. [12] increased the depth of CNNs and used BiLSTM to enhance signal time-domain connections. Kennedy et al. [13] used the random forest and K-approximation method to analyze the characteristics of RR interval. Kwang-Sig [14] compared the effects of AlexNet and ResNet in the classification of atrial fibrillation. Soliński et al. [15] use deep learning and hybrid QRS detection to classify atrial fibrillation signals. Gliner et al. [16] use a model composed of a support vector machine and a two-layer feedforward neural network to detect atrial fibrillation. Rubin et al. [17] first introduced densely connected convolutional networks in the classification of atrial fibrillation. Kumar et al. [18] use entropy features extracted from flexible analytic wavelet transform to detect the occurrence of atrial fibrillation. In the CinC 2017 competition, Zhao et al. [19] used Kalman filtering and Fourier transform to convert the ECG signals into a spectrogram and adopted an 18-layer deep neural network (DNNs) to extract and classify the features of the converted spectrogram. The final average F1 score on the test set is 0.802. Ping et al. [20] used a network model combining CNNs with jump connections and a long- and short-term memory neural network (LSTM) to classify ECG signals, and the F1 score in the test set was 0.896. Wu et al. [21] proposed a binarized convolutional neural network for the classification of atrial fibrillation signals, with an F1 score of 0.87 on the test set. [22] uses the shallow convolutional neural network and long short-term memory (LSTM) network. The addition of LSTM improves the classification accuracy. [23] uses time-frequency features to process the original data, and an artificial neural network (ANN) is used as feature extractors and classifiers. In literature [24], the authors use the squeeze-and-excitation residual network (SE-ResNet) to detect abnormal occurrence.

In this work, there is first time to use the dilated causal convolution in the ECG classification task. The main contributions are as follows:

1. A novel ECG classification method based on shortcut connection and dilated causal convolution is proposed. The proposed method effectively improves the training speed and classification accuracy
2. We explore the impact of the network structure and key parameters on classification results. A better parameter selection method is found, which further improved the classification accuracy of the model

The rest of the paper is organized as follows. In Section 2, the MIT-BIH AFDB [4] and the data preprocessing are described. In Section 3, the basic knowledge of DCC is introduced. In Section 4, the evaluation indicators of ECG signal classification and the experimental results are introduced. In Section 5, the summary of the whole paper is presented.

## 2. Database and Data Preprocessing

**Figure 1:**
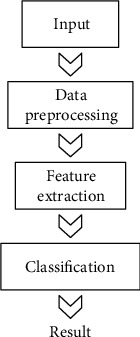
Four steps for intelligent classification of the ECG signal diagram.

The automatic classification of ECG signals is mainly divided into four steps: (1) input, (2) data preprocessing, (3) feature extraction, and (4) classification. The overall process is shown in Figure 1.

### 2.1. Database

The MIT-BIH AFDB [4] contains a total of 25 long-term ECG data, each record lasts for 10 hours, and the data sampling rate is 250 Hz. In addition, the data of No. 07735 and No. 03665 are not available. Therefore, the remaining 23 available records are used in experiments.

### 2.2. Data Preprocessing

#### 2.2.1. Denoising

There will inevitably be noise during ECG signal acquisition, so the DB6 wavelet is used to decompose the original ECG signal with a 9-level wavelet [25]. The components of the ECG signal are mainly concentrated between 0.05 and 40 Hz, so the first and second-level components containing 90-180 Hz and 45-90 Hz are discarded, and the remaining three to nine-level components are used for signal reconstruction.

#### 2.2.2. *Z*-Score Normalization

The amplitude of ECG data varies greatly among different people. When there are large differences in the input data, the performance of the neural network is often not good enough. Therefore, the *Z*-score normalization is adopted in data processing. This method reduces the impact of different amplitudes in the data. The process of *Z*-score normalization is carried out according to equation (1).

(1) $$\begin{array}{c} \text{Nor}\left(x\right)=\frac{x-\overset{¯}{x}}{\sigma }, \end{array}$$

where *x* is the ECG signal data, and $\overset{¯}{x}$ and *σ* are the average and standard deviation of the data.

#### 2.2.3. Segmentation

Since the length of the ECG data in the MIT-BIH AFDB is relatively long, the ECG data is segmented according to the label file to obtain 288 normal ECG data, 291 atrial fibrillation ECG data, and 14 atrial flutter ECG data. After segmentation according to the type, the obtained ECG signal is cut into segments with a length of 4 s. And the data with a length of less than 4 s is discarded. The data distribution after segmentation is shown in Table 1.

#### 2.2.4. 5-Fold Crossvalidation

**Figure 2:**
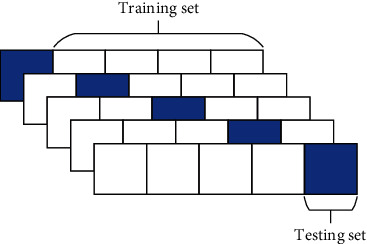
Data division diagram.

In the experiment, 5-fold crossvalidation is adopted. The experimental data are divided into five parts, of which four parts are used as the training set in turn and one part as the testing set. The 5-fold crossvalidation can improve the stability of the model and facilitate the selection of hyperparameters. The data division diagram is shown in Figure 2.

## 3. Method

In this section, in view of the slow operation speed of the traditional ECG classification model, the DCC is introduced in the automatic classification of ECG signals. To facilitate subsequent comparative experiments, Sections 3.1–3.3 introduce convolutional neural networks, recurrent neural networks, and time convolutional networks.

### 3.1. Convolutional Neural Networks (CNNs)

The convolutional layer is the core component of the convolutional neural networks (CNNs), in which most operations of convolutional neural networks are completed. The operation of the convolutional layer can be expressed by equation (2).

(2) $$\begin{array}{c} y=f\left(\overset{n}{\underset{i=1}{\sum }}\theta_{i}^{T}x_{i}+b\right), \end{array}$$

where *θ* is the weight parameter, *b* is the bias parameter, and *f*(·) represents the activation function.

The development of convolutional networks has gone through the stages of LeNet [26], AlexNet [2], VGGNet [27], and ResNet [28]. The potential of convolutional neural networks in feature extraction and classification have been continuously tapped. At the same time, the shortcomings of convolutional neural networks that cannot be well applied to time series information have been continuously amplified.

### 3.2. Recurrent Neural Networks (RNNs)

**Figure 3:**
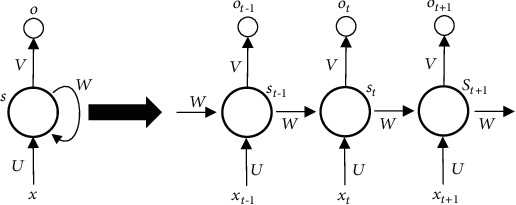
Recurrent neural network structure diagram [29].

Since convolutional neural networks cannot handle sequences related to time or space, recurrent neural networks (RNNs) [29] are proposed. The RNN network structure diagram is shown in Figure 3 [29], from which we can know that the structure diagram of RNNs is that the output value of the hidden layer of RNNs depends not only on the current input value but also on the output value of the hidden layer at the previous moment.

With the widespread application of RNNs models, the gradient problem in RNN networks has gradually attracted attention. At the same time, the shortcomings of the slow running time of RNN networks cannot meet people's needs.

### 3.3. Temporal Convolutional Network (TCN)

In order to solve the problems of RNNs, Bai et al. [30] proposed a temporal convolutional network (TCN) to process time series information. TCN is a network structure based on the CNN network framework to achieve similar functions of the RNN network. To solve the problem of different input and output time steps in CNNs and future information leakage, TCN was proposed.

The dilated causal convolutional layer is the core network layer of the TCN. DCC can be divided into two parts: dilated convolution [31] and causal convolution [32]. Causal convolution can solve the problem of different input and output time steps in the CNNs model and future information leakage. Dilated convolution can widen the receptive field of the convolution kernel and reduce the network depth to a certain extent.

### 3.4. Improved Dilated Causal Convolutional Network

**Figure 4:**
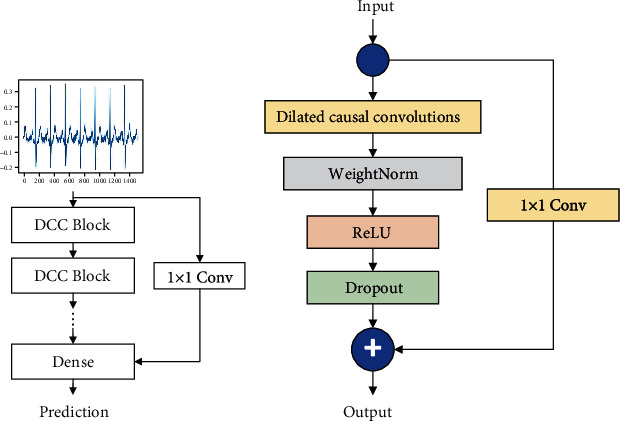
(a) iDCC Network structure. (b) DCC Block composition structure.

The ECG signals are time series, and the length is relatively long. These features can match the advantages of TCN. However, the result of the experiment is not satisfactory. To obtain better results, we propose an improved model. Improved model contains multiple DCC blocks and multiple shortcut connections [28]. In the proposed model, each block contains a dilated causal convolutional layer, a weight normalization layer, an activation function layer, a dropout layer, and a shortcut connection. And we also added a shortcut connection layer between the input layer and the fully connected layer.  Figure 4 shows the structure of the proposed model.

#### 3.4.1. Causal Convolution

**Figure 5:**
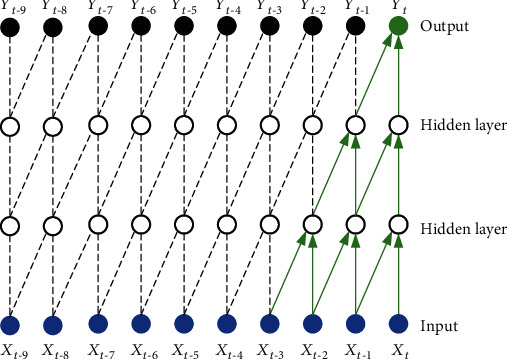
Diagram of causal convolution [32].

To solve the problem of information leakage in the future, casual convolution [32] is adopted in the model. For the output data *yt* at time *t*, the input can only be *t* and the time before *t*; that is, *x*0, *x*1 ⋯ *xt* and its structure diagram are shown in Figure 5.

#### 3.4.2. Dilated Causal Convolution

**Figure 6:**
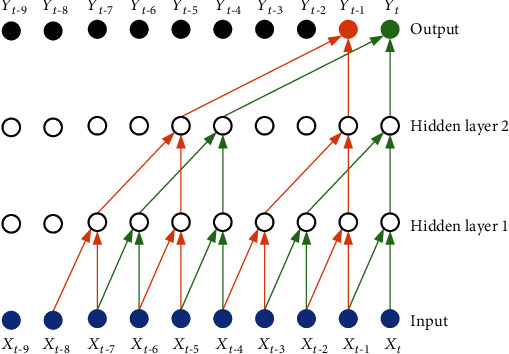
Diagram of dilated causal convolution [30].

**Figure 7:**
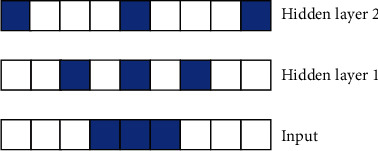
Visualization of the 1D convolution kernel with different dilation factors.

Since the ECG signal generally has a high sampling rate and the collected signal lasts for a long time, the direct use of causal convolution will cause the network layer to be too deep, which is not conducive to neural network learning and greatly increases the computational burden. In order to effectively deal with data with long historical information such as ECG data, the idea of WaveNet [33] and dilated causal convolution (DCC) are introduced into the model. The dilated factor *d* [34]is introduced on the basis of causal convolution, which increases the size of the receptive field and can reduce the number of network layers to a certain extent. The diagram of the DCC operation is shown in Figure 6 [30].  Figure 7 shows the 1D convolution kernel with the convolution factor added.

#### 3.4.3. Weight Normalization

To further speed up the network operation, we changed the standardization layer in the model from the batch normalization layer to the weight normalization (WN) [35] layer. The operation of the neural network can be expressed by equation (3).

(3) $$\begin{array}{c} y=\phi \left(\omega \cdot x+b\right), \end{array}$$

where *ω* is the feature vector. The normalization strategy proposed by WN is to decompose *ω* into a parameter vector *v* and a parameter scalar *g*. The decomposition method is shown in equation (4) [35].

(4) $$\begin{array}{c} \omega =\frac{g}{\left\|v\right\|}v. \end{array}$$

In the above formula, ‖*v*‖ represents the Euclidean distance of *v*. The updated values of *v* and *g* can be calculated by SGD [36]. Equation (5) [36] and equation (6) [36] show the calculation process.

(5) $$\begin{array}{c} \nabla_{g}L=\frac{\nabla_{w}L\cdot v}{||v||}, \end{array}$$

(6) $$\begin{array}{c} \nabla_{v}L=\frac{g}{||v||}\nabla_{w}L-\frac{g\nabla_{g}L}{||v{||}^{2}}v, \end{array}$$

Where *L* is the loss function, and ∇_*w*_*L* is the gradient value of *w* under *L*.

#### 3.4.4. Activation Function

The ReLU [37] activation function is applied in the model. Equation (7) [37] shows the calculation process of the ReLU activation function.

(7) $$\begin{array}{c} \text{ReLU}\left(x\right)=\max\left(0,x\right). \end{array}$$

#### 3.4.5. Dropout Layer

To prevent the model from overfitting, a dropout layer [38] is added to the model. The operational expression of the dropout layer is shown in equation (8) [38].

(8) $$\begin{array}{c} r_{j}^{\left(l\right)}∼\text{Bernoulli}\left(p\right),\\ {\overset{˜}{\text{y}}}^{\left(l\right)}=r^{\left(l\right)}\times y^{\left(l\right)},\\ z_{i}^{\left(l+1\right)}=w_{i}^{\left(l+1\right)}{\overset{˜}{y}}^{l}+b_{i}^{\left(l+1\right)},\\ y_{i}^{\left(l+1\right)}=f\left(z_{i}^{\left(l+1\right)}\right). \end{array}$$

In the above formula, the Bernoulli function will randomly generate a vector of 0 or 1.

#### 3.4.6. Shortcut Connections

**Figure 8:**
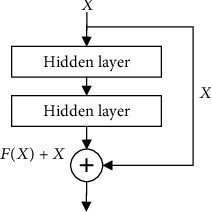
Diagram of shortcut connection [28].

The residual block structure usually appears in neural networks with deeper network structures. He [28] showed in the research that when the network depth reaches a certain level, continuing to increase, the network depth will make the learning effect worse. The residual network makes the network easier to optimize by adding shortcut connections to the deep neural network. Several layers of networks containing a short connection are called a residual block, as shown in Figure 8. The calculation expression of the shortcut connection is shown in equation (9) [28].

(9) $$\begin{array}{c} o=\left(X+F\left(X\right)\right). \end{array}$$

**Figure 9:**
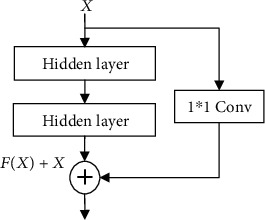
A convolution block joined in the shortcut connection [28].

The number of channels between the original data *X* and the data *F*(*X*) after the convolution operation may not be equal. Therefore, a 1 × 1 convolution block is added to the jump connection to perform a simple transformation on *X*, so that the transformed *X* and *F*(*X*) have the same number of channels. The structure is shown in Figure 9 [28].

## 4. Experiment and Results

The network structures proposed in this article are built by the PyTorch framework and trained on Nvidia Tesla V100 GPU. The Adam [39] optimization algorithm is used for training, the initial value of the learning rate is set to 0.0001, and the Cosine Annealing [40] is adopted. The number of iteration rounds is set to 50.

### 4.1. Evaluation Index

Accuracy (Acc), specificity (Spe), and sensitivity (Sen) are three important evaluation indicators of neural network models. To calculate these evaluation indicators, the true positive (TP), true negative (TN), false positive (FP), and false negative (FN) are introduced. The calculation equations of the evaluation indexes are shown in equations (10)–(12).

(10) $$\begin{array}{c} \text{Acc}=\frac{\text{TP}+\text{TN}}{\text{TP}+\text{TN}+\text{FP}+\text{FN}}, \end{array}$$

(11) $$\begin{array}{c} \text{Spe}=\frac{\text{TN}}{\text{TN}+\text{FP}}, \end{array}$$

(12) $$\begin{array}{c} \text{Sen}=\frac{\text{TP}}{\text{TP}+\text{FN}}. \end{array}$$

### 4.2. Experimental Verification

#### 4.2.1. Accuracy Comparison Experiment

**Figure 10:**
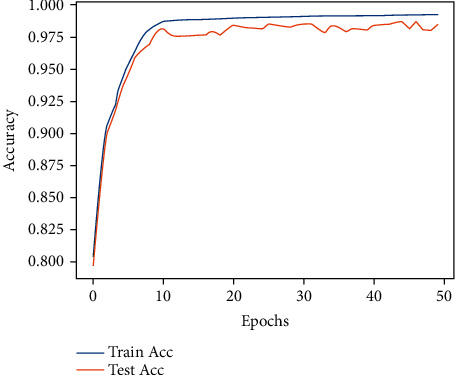
Accuracy of iDCCN classification results.

**Figure 11:**
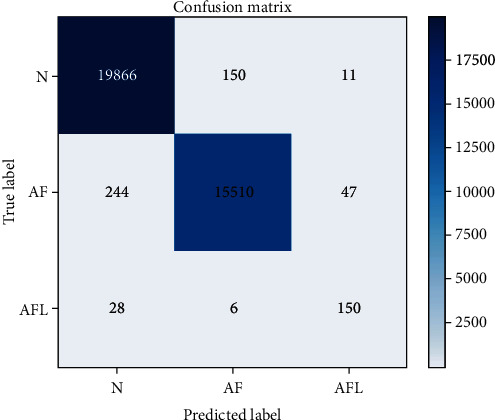
Confusion matrix of iDCCN classification results.

The accuracy of the improved dilated causal convolutional neural network (iDCCN) in the training set and the testing set of the atrial fibrillation database is shown in Figure 10, and the confusion matrix of the classification results in the testing set in Figure 11. The classification accuracy (Acc) of iDCCN in the MIT-BIH AFDB is 98.65%, the sensitivity is 98.79%, and the specificity is 99.04%.

Table 2 summarizes several classification algorithms that have performed well in the MIT-BIH AFDB in recent years. The table lists the author of the method, the year of publication, the method used, and the performance of the method in the database. [7] is based on the shape of the ECG signals, and the classification effect will decrease when the signals type is more complex. [8, 9] are machine learning methods, which requires a large number of manual features extracted as well as high data preprocessing results when dealing with problems, taking a lot of time and computation. [10] uses an 11-layer convolutional neural network to detect the occurrence of atrial fibrillation. This method achieves the accuracy of 94.9%, the sensitivity of 99.13%, and the specificity of 81.44%. However, the network model of this method is relatively simple, and the classification results are not ideal. The method mentioned in [11] achieves the accuracy of 97.80%, the sensitivity of 98.96%, and the specificity of 86.04%. However, the complex networks slow down the calculation speed, and the classification result is also very dependent on the detection result of the RR interval. The method of [12] has reached 96.59%, 99.93%, and 97.03% in accuracy, sensitivity, and specificity, respectively. However, due to the deeper network depth and the use of LSTM, the network has a large amount of calculation and slower computation speed.

#### 4.2.2. Complexity Analysis

To verify the superiority of the proposed method in running time, we reproduced the network model used in [10–12, 22–24] and recorded the running time of the model on the testing set.  Table 3 shows the running time of the four different models in the testing set.

As shown in Table 3, the network used in [10] costs the least time in the testing set, with a duration of 25.67 s, but due to the simple network structure, the classification accuracy is low. Since the model network used in [11] has a deeper number of layers, it takes a long time in the testing set, with a duration of 32.60s, but the accuracy has been improved. The network used in [12] has a more complex network structure and deeper network depth. It spends longer time on the testing set, with a duration of 40.82 s. In literature [22], the addition of LSTM improves the classification accuracy. However, the duration is 30.64 s on the testing set. [23] gets the highest classification accuracy in the comparative experiments. But due to the complex network structure, [23] takes the longest time in the testing set, with a duration of 48.37 s. And because of the deeper network depth, the running time of [24] on the testing set is 46.20s.

The proposed method removes the recurrent neural network in the model, which reduces the overall time complexity. The running time on the testing set is 27.62 s. And in traditional convolution layers, convolution kernels are tightly connected. But in the proposed model, the addition of dilated factors reduces the computational complexity of the convolutional layer.

#### 4.2.3. Network Structure Comparison Experiment

To verify whether the number of dilated causal convolution blocks affects the experimental results, 3 blocks, 4 blocks, and 5 blocks are used relatively for comparison, and four different ways are adopted to define the dilated factor.

1. *d* = 0
2. *d* = *i*
3. *d* = 2 × (*i* + 1)
4. *d* = 2^*i*^where *d* is the dilated factor. *i* is the block number. *i* starts from 0.

**Figure 12:**
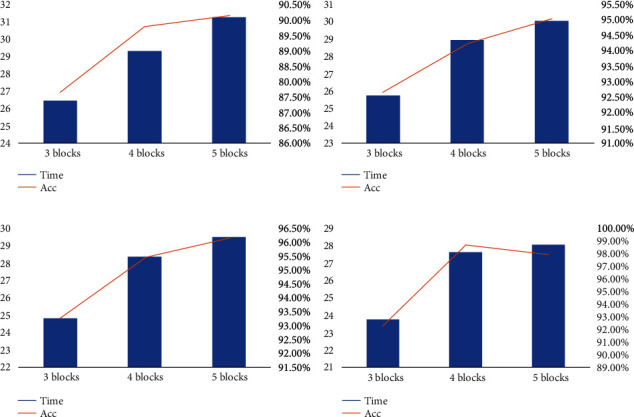
Comparison of time and accuracy under different numbers of blocks. (a) *d* = 0, (b) *d* = *i*, (c) *d* = 2 × (*i* + 1), (d) *d* = 2^*i*^.

As shown in Table 4 and Figure 12, with the increase of network depth, the amount of computation is increasing. In the same computing capability, increased amount of computation means increase in computation time. Also, with the increase of network depth, the learning ability of the model is enhanced, and the classification accuracy is improved.

When the dilated factor is 0, the computation time of 3 blocks, 4 blocks, 5 blocks is 26.48 s, 29.34 s, 31.26 s, respectively. And the accuracy is 87.66%, 89.78%, 90.14%. In the second case, the dilated factor is *i* (*i* is block number). The computation time of 3 blocks, 4 blocks, 5 blocks is 25.76 s, 28.97 s, 30.06 s, respectively. The accuracy is 92.65%, 94.22%, 95.03%. In the third case, the dilated factor is 2∗(*i* + 1). 24.83 s, 28.35 s, 29.51 s is the computation time of 3 blocks, 4 blocks, 5 blocks. And the accuracy of three experiments is 93.27%, 95.43% and 96.15%. In the last case, the dilated factor is 2^*i*^. The computation time of three experiments is 23.76 s, 27.62 s, 28.06 s. The accuracy is 92.31%, 98.65%, 97.92%.

The accuracy reaches the highest in the last case when the number of blocks is 4. And in the last case, the accuracy curve first rises in 3 and 4 block experiments and then falls in 5 block experiments. This may be caused by the network falling into a local optimal solution.

## 5. Conclusion

This paper proposes a novel ECG signal classification model based on DCC. The proposed model contains four iDCCN blocks, and each iDCCN block contains a dilated causal convolutional layer, a weight normalization layer, an activation function layer, a dropout layer, and a shortcut layer. 5-fold crossvalidations are used to train and test the model on the MIT-BIH AFDB. The proposed model increases the classification accuracy to 98.65% in the testing set. Experimental results validate the effectiveness of this method in atrial fibrillation detection. And the model reduces the running time. The method provides new ideas for real-time diagnosis of ECG signals.

## Figures and Tables

**Table 1:** Data distribution of MIT-BIH AFDB.

Type	Num
Normal	124808
Atrial fibrillation (AF)	83626
Atrial flutter (AFL)	1449
Total	209883

**Table 2:** Summary of selected studies conducted for the automated detection of AF.

Author, year	Method	Performance		
		Acc	Sen	Spe
Lake et al., 2011 [7]	Coefficient of sample entropy (COSEN)	—	91%	98%
Asgari et al., 2015 [8]	Stationary wavelet transforms, support vector machine (SVM)	_—	97%	97.1%
Zhou et al., 2014 [9]	Recursive algorithms	97.67%	96.89%	98.25%
Acharya et al., 2017 [10]	CNNs	94.9%	99.13%	81.44%
Andersen et al., 2018 [11]	CNNs-RNNs	97.80%	98.96%	86.04%
Dang et al., 2019 [12]	Deep CNN-BiLSTM	96.59%	99.93%	97.03%
Proposed	iDCCN	98.65%	98.79%	99.04%

**Table 3:** Complexity analysis of the seven models in the testing set.

Model	Time(s)	Accuracy
Acharya et al., 2017 [10]	25.67	94.93%
Andersen et al., 2018 [11]	32.60	97.80%
Dang et al., 2019 [12]	40.82	96.59%
Ma et al., 2020 [22]	30.64	97.21%
Sangaiah et al., 2020 [23]	48.37	99.11%
Park et al., 2020 [24]	46.20	97.05%
Proposed	27.62	98.65%

**Table 4:** Comparison of time and accuracy under different numbers of blocks. (A) *d* = 0, (B) *d* = *i*, (C) *d* = 2 × (*i* + 1), and (D) *d* = 2^*i*^.

	(A)		(B)		(C)		(D)	
	Time(s)	Acc	Time(s)	Acc	Time(s)	Acc	Time(s)	Acc
3 blocks	26.48	87.66%	25.76	92.65%	24.83	93.27%	23.76	92.31%
4 blocks	29.34	89.78%	28.97	94.22%	28.35	95.43%	27.62	98.65%
5 blocks	31.26	90.14%	30.06	95.03%	29.51	96.15%	28.06	97.92%

## Data Availability

The ECG signal data used to support the findings of this study have been deposited in the MIT-BIH Atrial Fibrillation Database repository (https://www.physionet.org/content/afdb/1.0.0/).
